# Surgical procedure of segmentectomy as a possible cause of postoperative cerebral embolism: a case report

**DOI:** 10.1186/s13019-020-01378-7

**Published:** 2020-12-14

**Authors:** Peirui Chen, Qiusha Qing, Mingqiang Diao, Xiaokang Sun, Junrong Yang, Jing Lv

**Affiliations:** 1 Department of Cardiothoracic Surgery, People’s Hospital of Deyangcity, Deyang, China; 2 Department of Imaging, People’s Hospital of Deyangcity, Deyang, China

**Keywords:** Cerebral embolism, Segmentectomy, Lung cancer

## Abstract

**Background:**

Cerebral embolism after lobectomy is a life-threatening complication during the early postoperative period. However, it is unclear if cerebral embolism can develop after segmentectomy.

**Case presentation:**

We experienced a case of a 37-year-old man who demonstrated early symptom of acute ischemic stroke in early postoperative period after right upper posterior segmentectomy and performed intra-arterial mechanical thrombectomy (IAMT) successfully.

**Conclusions:**

Long and irregular pulmonary vein stump (PVS) and endothelial injury caused by surgical procedure may lead to cerebral embolism after segmentectomy. We believe that doing preoperative pulmonary vascular assessment and using appropriate surgical procedure may reduce the rate of cerebral embolism.

## Background

Cerebral embolism is an uncommon and serious complication during the early postoperative period after lung cancer surgery [1]. Sublobar resection (segmentectomy or wedge resection) has been recommended as an important treatment for cases of small-sized non-small cell lung carcinoma (NSCLC) [2]. Although some cases of cerebral embolism associated with lobectomy have been reported so far [1, 3, 4], cerebral embolism caused by a thrombus in the vein stump after right upper posterior segmentectomy has not been reported to our knowledge.

We experienced a case of a patient with cerebral embolism in the first postoperative day, and performed intra-arterial mechanical thrombectomy (IAMT) successfully.

## Case presentation

**Fig. 1:**
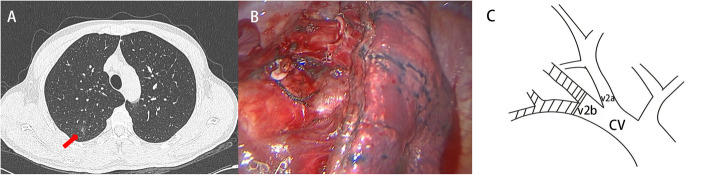
**a** An 8-mm solitary ground-glass opacity lesion is identified at the right S^2^ with coronal views on computed tomography scanning (arrow). **b** The VATS right S^2^ segmentectomy operation field. **c** A diagram of the venous anatomy (The shaded part represents the draining veins of posterior segment)

A 37-year-old man who was 172 cm tall and weighed 62 kg, BMI 21.5 kg/m^2^ underwent video-assisted thoracoscopic right upper posterior segmentectomy with systematic mediastinal lymphadenectomy. His had no history or ongoing (chronic) health problems like hypertension, diabetes mellitus, cerebrovascular disease and atrial fibrillation (AF). He had a smoking history of 15 pack-years. A an 8-mm ground-glass opacity (GGO) lesion had been identifed at the posterior segment (S2) of the right upper lobe during incidental computed tomography (CT) screening 6 months prior to his presentation to us. The ground-glass nodules grew slightly during the follow-up period (Fig. 1a). Results of all preoperative laboratory tests including electrocardiogram, blood examination and urine examination were within normal limits. Platelet function tests were not performed.

During the surgery, the patient was placed in the left lateral decubitus position with the arms extended to 90°. General anesthesia was induced, and intubation was achieved via a double-lumen endobronchial tube. The first stage of this operation was opening the oblique fissure. Incomplete oblique fissure was divided via endoscopic stapler (Ethicon ECHELON FLEX™ ENDOPATH EC60A stapler, blue staples). We exposed the central vein, V^2a^ and V^2b^ but not found and isolated the draining veins of posterior segment (S^2^) specifically (Fig. 1b). Next the pulmonary artery was dissected distal to expose the posterior segmental artery(A [2]). The A^2^ was then dissected, and ligated by hemolok. Then the posterior segmental bronchus was dissected from right superior lobar bronchus. Before closing the bronchus with the stapler, we ventilated the right lung to confirm the target bronchus. The B^2^ was then transected by stapler (Ethicon ECHELON FLEX™ ENDOPATH EC60A stapler, green staples). Then, the S^2^ parenchyma was divided followed the inflation-deflation method by stapler (Ethicon ECHELON FLEX™ ENDOPATH EC60A stapler, blue staples) [5]. The specimen was retrieved in a self-made bag using a surgical glove. Examination of a frozen section confirmed lung adenocarcinoma; Systematic mediastinal lymphadenectomy was then performed. The total operation time was 1 h, 15 min. The postoperation pathological diagnosis of the tumor was adenocarcinoma (pT1aN0M0-stage IA1 as defined by the 8th Edition of TNM classification (TNM8) of lung cancer) [6].

**Fig. 2:**
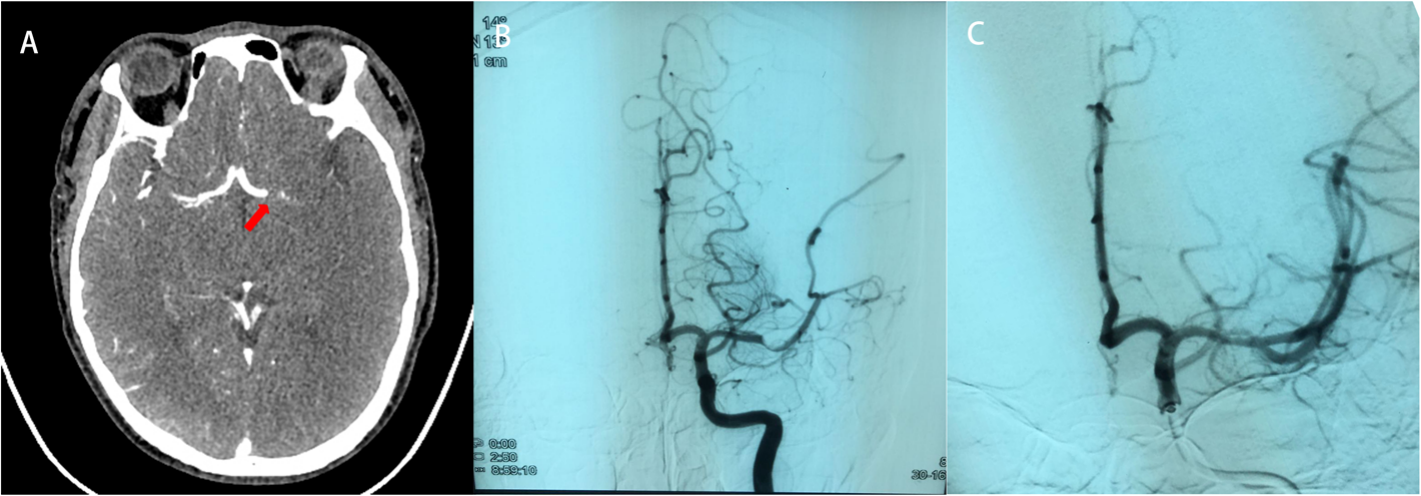
Cerebral embolism in the region of the left middle cerebral artery. **a** Head computed tomography displayed left middle cerebral artery M1 occlusion (arrow). **b** Angiography showing left middle cerebral artery occlusion. **c** The recanalization of intra-arterial mechanical thrombectomy (IAMT) in Cerebral

**Fig. 3:**
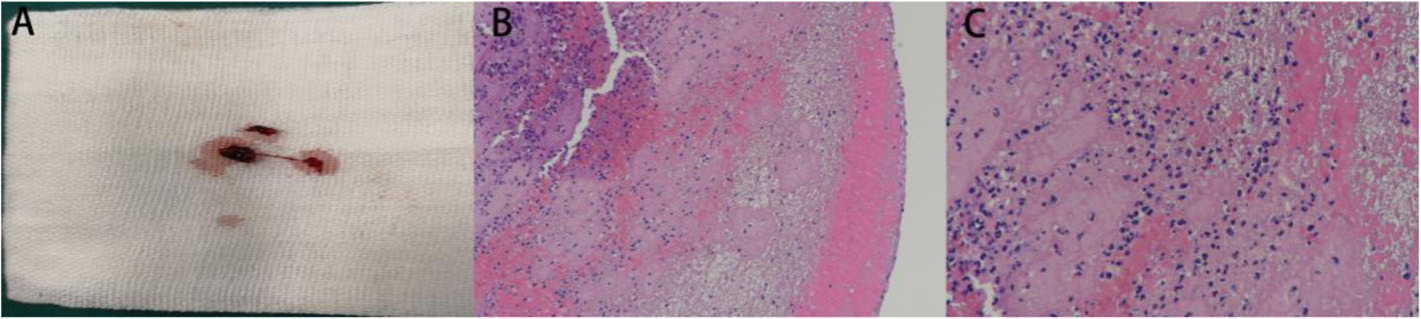
Gross appearance and histopathology of the removed thrombus. **a** Gross appearance of the removed thrombus. **b** The thrombus is composed of neutrophils, fibrin, platelets and red blood cells. **c** Many neutrophils trapped within a fibrin network. Hematoxylin–eosin stain. [original magnification × 100 (**b**); original magnification × 400 (**c**)]

After surgery the patient returned to the intensive care unit. 20 h later, Mild consciousness disturbance [Glasgow Coma Scale, 11 points (E3V2M6)], conjugate deviation of the eyes to the left, right hemiplegia (Manual Muscle Testing upper limbs score of 1 of 5 and lower limbs score of 0 of 5), mixed aphasia were observed, with National Institutes of Health Stroke Scale score of 23. His vital signs were a blood pressure of 118/67 mmHg, a heart rate of 107 /min, a respiration rate of 17 /min and SpO2 of 100% with nasal catheter oxygen at 4 L/min. Emergent head enhanced CT showed left middle cerebral artery M1 occlusion (Fig. 2a). The left middle cerebral artery were not visualized in intracranial vascular computed tomography angiography (CTA). Considered the risk of hemorrhage in the postoperative period, we decided to perform IAMT. Trombus at the M1 branch of the middle cerebral artery were removed successfully, and successful recanalization was achieved (Fig. 2b, c). A pathological examination after the operation showed the thrombus were mainly composed of laminar fibrin, abundant neutrophils, and erythrocytes (Fig. 3).

The patient was observed in the intensive care unit for 3 days. The patient’s Mental status and motor function gradually improved. During this time, We performed some physiological examinations including Holter electrocardiography, transthoracic echocardiography, venous ultrasonography, carotid artery ultrasonography, and also checked blood tests such as coagulation function, and autoantibodies of collagen diseases. However, no anatomic abnormality or thrombophilia factors were observed. Three days after thrombus removal, his NIHSS score was 0. Follow up brain CT were done, and there was no significant findings. The patient was discharged without major complications after rehabilitation training for 7 days.

## Discussion and conclusions

Although lobotomy is still recommended as a standard surgical procedure for lung cancer, segmentectomy that reveal farthest excision of pulmonary tumor and farthest preservation of normal lung tissues, has extended its surgical indications to some early stage lung cancer. Multi-center retrospective study found that the curative effect of segmentectomy for stage Ia lung cancer is similar to lobotomy, with no difference in recurrence rate and 5-year survival rate [7–9]. it also be associated with fewer postoperative complications and better lung function [10, 11]. Cerebral embolism is a serious complication in the early postoperative period, occurring in 0.2–1.2% of surgical lung cancer cases [12]. In this case, we present an rare case of cerebral embolism after segmentectomy. There was only one risk factor for cerebrovascular disease (long-term smoking history) before surgery, and relevant preoperative examination did not indicate the risk of cerebral embolism (AF and Hypercoagulability). Electrocardiograph monitoring was also used all through the the first postoperative day, later a Holter electrocardiography was performed. We found no AF in the postoperative period. So we highly suspicious cerebral embolism was caused by a thrombus in the PVS because there were no other suspected sources.

Pulmonary vein directly connects to the left cardiac system. Because of this anatomical feature, thrombus in the PVS may leads to a cerebral thrombosis. Left upper lobectomy more likely to results in a longer pulmonary vein stump, which provides an explanation for the higher incidence of cerebral embolism after left upper lobectomy than after other types of lobectomy [3, 13, 14]. Those researches indicated that the length of the PVS is an important factor affecting thrombus formation. In 2015, The pulmonary vein branching pattern of right upper lung was classified into four types by three-dimensional CT angiography [15]. In this case, besides V^2a^ and V^2b^, we did not find the independent draining veins of posterior segment (S2). Thus we suppose that it belongs to “anterior with central vein type, I_ab_ type”, which V^1a^ and V^1b^ drained into V. ant, V^2a^ and V^2b^ drained into V. cent. This variation is the most common pattern of right upper lung and present in 54 -83.2% of patients [15, 16]. To furthest preserve venous drainage of the residual segment, we didn’t ligate the V^2a^ and V^2b^. However the draining veins of posterior segment (S^2^) must have been divided at the time of dividing the parenchyma. A diagram of the venous anatomy is made according to the CT scan and intraoperative findings (Fig. 1c). The PVS was thick enough for chunky thrombosis. However, because no lung enhanced CT scan was performed before and after surgery, how veins were divided is not exactly clear. So we consider this blind procedure might leave a long and irregular PVS. Furthermore, the intersegmental veins in separating intersegmental plane might also be injured by stapler.

Previous researches indicated that endothelial injury and immune cells plays an important role in thrombogenesis in the PVS [4, 12]. In our case, after dividing the draining veins with stapler, interlocking nails stabed into the endothelium and leaved behind in the tissue of PVS. Endothelial injury consequently activated the extrinsic pathway of the coagulation cascade and resulted in thrombus formation in the PVS. The pathological examination of the removed thrombus is composed of fibrin along with abundant neutrophils and erythrocytes, which also suggesting a inflammatory response caused by tissue injury (Fig. 3).

According to our case, the surgical procedure seems to be an important factor for cerebral embolism after segmentectomy. We suggested that before divide the veins of posterior segment, the branches of the central vein should be cautiously isolated, with the subsegmental veins kept intact. In addition, we recommend using silk thread or hemolok instead of using surgical stapling device to divide the veins. Furthermore several researches have demonstrated that preoperative computed tomography 3-dimensional (3D) reconstruction helps in asserting the number, size, and direction of vessels [17–19]. Checking the preoperative 3D model before operation may help the surgeon to perform a more accurately and safer dissection of the branches of the pulmonary vessels.

In conclusion, this is the first case of a cerebral embolism presumably caused by a thrombus in the PVS after segmentectomy for lung cancer. The thrombus might have been formed in the PVS due to the long and irregular PVS and tissue injury caused by surgical stapling device in the early postoperative period.

## Data Availability

The patient’s file is under the possession of people’s hospital of deyangcity. The datasets used and/or analyzed during the current study are available from the authors on reasonable request.

## Abbreviations

PVS: Pulmonary vein stump
IAMT: Intra-arterial mechanical thrombectomy
NSCLC: Non-small cell lung carcinoma
AF: Atrial fibrillation
GGO: Ground-glass opacity
